# Reliability of Questionnaires to Assess the Healthy Eating and Activity Environment of a Child's Home and School

**DOI:** 10.1155/2013/720368

**Published:** 2013-07-01

**Authors:** Annabelle Wilson, Anthea Magarey, Nadia Mastersson

**Affiliations:** ^1^Nutrition and Dietetics, Flinders University of South Australia, GPO Box 2100, Adelaide, SA 5001, Australia; ^2^Discipline of Public Health, Flinders University of South Australia, GPO Box 2100, Adelaide, SA 5001, Australia; ^3^Health Promotion Branch, SA Health, Government of South Australia, P.O. Box 287 Adelaide, SA 5000, Australia

## Abstract

Childhood overweight and obesity are a growing concern globally, and environments, including the home and school, can contribute to this epidemic. This paper assesses the reliability of two questionnaires (parent and teacher) used in the evaluation of a community-based childhood obesity prevention intervention, the *eat well be active (ewba)* Community Programs. Parents and teachers were recruited from two primary schools and they completed the same questionnaire twice in 2008 and 2009. Data from both questionnaires were classified into outcomes relevant to healthy eating and activity, and target outcomes, based on the goals of the *ewba* Community Programs, were identified. Fourteen and 12 outcomes were developed from the parent and teacher questionnaires, respectively. Sixty parents and 28 teachers participated in the reliability study. Intraclass correlation coefficients for outcomes ranged from 0.37 to 0.92 (parent) (*P* < 0.05) and from 0.42 to 0.86 (teacher) (*P* < 0.05). Internal consistency, measured by Cronbach's alpha, of teacher scores ranged from 0.11 to 0.91 and 0.13 to 0.78 for scores from the parent questionnaire. The parent and teacher questionnaires are moderately reliable tools for simultaneously assessing child intakes, environments, attitudes, and knowledge associated with healthy eating and physical activity in the home and school and may be useful for evaluation of similar programs.

## 1. Introduction

Overweight and obesity are a global concern in both developed and developing countries and in school age children, the prevalence continues to remain high. Australian childhood overweight and obesity rates have been shown to be 25.3% for boys and girls aged 5–17 years, comprised of 17.7% overweight and 7.6% obese [1]. There is a clear need for effective prevention efforts to address the high prevalence of childhood and adult obesity [2] without which obesity will become the primary cause of preventative deaths worldwide [3].

Management of this epidemic requires action at a number of levels including broad-based community interventions that focus on environmental change to support individual behaviours [4–6]. Governments need to contribute to less obesogenic environments through political, physical, sociocultural and structural change, and prevention programs [2, 7, 8]. Despite a large body of the literature pertaining to the management of childhood obesity, there are a limited but increasing number of community-wide prevention projects. To effectively target childhood obesity, existing and new programs need to be systematically evaluated to determine the efficacy of the implemented strategies and such evaluations should be of high quality in order to contribute to the evidence for addressing childhood obesity [9]. However, these evaluations are limited by a lack of setting specific tools which allow evaluation specific to a particular setting, such as a school or home environment [10], and further limited by a lack of reliable tools suitable for evaluation purposes in these settings. 

The *eat well be active* (*ewba*) Community Programs were implemented in South Australia from 2005 to 2010, focusing on prevention of obesity through environmental change using a community development approach. Details of this program have been published [10–15]. Briefly, the program aimed to increase the proportion of 0–18 year old children within the healthy weight range by effecting environmental change which in turn would influence healthy eating and physical activity behaviours. A comprehensive evaluation framework was included in the program [12]. Results from the evaluation of *ewba *have been reported elsewhere [12–15].

Due to the lack of relevant tools to evaluate the impact of the intervention, a number of program-specific questionnaires were developed to assess behaviours, knowledge, attitudes, and environments relevant to the goals of the program of increasing healthy eating and activity. Four of these tools were completed by children aged 9 to 11 years, their parents, and teachers. A critical factor in the use of any tool is its reliability. Assessment of these tool properties is important to ensure that accurate and appropriate assessments can be conducted [16]. The reliability and internal validity of the Child Nutrition Questionnaire have been reported [10].

The aim of this paper is to report the reliability of the parent and teacher questionnaires, tools that assess the diet and physical activity environments of children in the home and school, respectively. These questionnaires can provide relevant insight into the domains which influence nutrition and physical activity behaviours in children, and may be used to evaluate obesity prevention interventions.

## 2. Methods

Ethics approval for this study was obtained from the Flinders University Social and Behavioural Research Ethics Committee, the South Australian Health Ethics Committee, and the Department of Education and Children's Services Ethics Committee. 

### 2.1. Subjects

Subjects were recruited from two schools. School 1 was a reception-Year 12 (5–18 years) government school in the north of Adelaide and School 2 a Reception-Year 12 Catholic school in a regional centre in South Australia. School 2 was part of the wider *ewba* evaluation, but School 1 was not. All parents of children in years 5–7 at School 1 were invited to participate in the test-retest reliability study, and all teachers were invited at a staff meeting, in October 2008. At School 2, the test-retest reliability study was conducted in conjunction with the *ewba* follow-up evaluation. Parents who participated in this *ewba *evaluation follow-up (Sept–Nov 2009) were invited by letter to complete the questionnaire on a second occasion, and all teachers were asked at a staff meeting to complete the survey a second time. 

At School 1, Test 1 was administered to all teachers at a staff meeting. At School 2, Test 1 was administered to teachers who agreed to participate whose classes were participating in the wider *ewba *evaluation. At School 1, the parent questionnaires were sent home by the school to all parents of students in school years five, six, and seven. An introductory letter, an information sheet, and reply-paid envelope to allow return by post accompanied the questionnaire. At School 2, the parent questionnaires were administered as part of the wider *ewba* evaluation, as reported in [11]. Two weeks later at both schools, teachers completed the questionnaire again at a staff meeting, and parents who completed the first questionnaire were mailed the second questionnaire with a reply-paid envelope. Reminder letters were sent two weeks later by the school to parents who had not returned the second questionnaire. Both questionnaires took approximately 20 minutes to complete. 

### 2.2. Development of Questionnaires

The parent and teacher questionnaires were part of a suite of questionnaires developed for evaluation of the *ewba *Community Programs. The questionnaires were developed by the program evaluation committee which included academics with expertise in childhood obesity, nutrition, physical activity, and community development. These were developed in response to a lack of valid and relevant tools being available in the published or the grey literature. The items included in the program-specific questionnaires were specifically selected to evaluate each of the program's relevant objectives. Thus, these questionnaires were likely to be more sensitive to the programs' goals and objectives than any existing questionnaires which were more general and did not include the breadth of the programs' inquiry.

The parent questionnaire contains 25 questions requiring 67 responses covering the following domains: demographics; obesogenicity of the home environment; parental knowledge and attitudes towards healthy eating and physical activity; child physical activity and healthy eating behaviours. Two additional questions ask about food security and difficulty of breastfeeding in public places (Table 1). The teacher questionnaire consists of 15 questions requiring 44 responses covering teaching practices around healthy eating and physical activity inclusion in the school curriculum; training/experience in healthy eating and physical activity; teacher knowledge and attitudes towards healthy eating and physical activity (Table 2).

### 2.3. Scoring the Questionnaires

To produce more meaningful and reliable results, some responses are condensed into “scores” by summing items which represent a specific domain. This is one way of interpreting information obtained from dietary indexes and has been used previously [10, 17–19]. In order to make the analyses specific to *ewba *and to be able to measure the impact of the intervention directly by relating to the specific project goals, items were grouped into scores based on the goals of the *ewba *Community Programs. For example, goals related to diet included decreasing child intake of sweetened beverages and noncore foods and increasing intake of water and fruit and vegetables; hence, these were used as categories for the basis of developing scores related to child intake. Similarly, goals related to physical activity were increasing active pastimes and decreasing sedentary activity; hence, these were used as the basis for classifying items into scores demonstrating child physical activity. Generally, *ewba *sought to influence at multiple levels including attitudes, behaviours, and environments; therefore, scores assessed one of these three domains in the context of the diet or activity factors mentioned previously (non-core food, sweetened beverages, water, fruit and vegetables, active pastimes, and sedentary pastimes). Items were reverse coded where necessary, so a higher score represented a more positive attitude or behaviour. The created scores allow better assessment of health themes, enablers, and barriers and can be used to compare baseline data against a “target” score. Outcomes that could not be grouped into a score are left as single items. Thus, there are 14 outcomes (seven scores and seven single items) from the parent questionnaire and 12 outcomes (six scores and six single items) from the teacher questionnaire. A target score which identifies a healthy attitude or environment was determined for each score (Tables 3 and 4). Targets for assessment of intake were largely based on the Australian Guide to Healthy Eating (AGHE) [20]. Assessment of physical activity was based on the Physical Activity Guidelines for five to 12 year olds [21] and 12 to 18 year olds [22]. 

### 2.4. Statistical Analysis

All data were analysed using SPSS 18.0.1. (SPSS Inc.) Statistical significance was accepted at *P* ≤ 0.05. The 14 parent and 12 teacher outcomes were assessed for test-retest reliability using the intraclass correlation coefficient (ICC). Internal consistency of the teacher and parent scores (outcomes with multiple items; parent questionnaire seven scores; teacher questionnaire 6 scores) at Time 1 was assessed using Cronbach's alpha. 

## 3. Results

### 3.1. Parent Questionnaire Test-Retest Reliability

Sixty parents (School 1: 22, School 2: 38) completed the questionnaire on two occasions one to two weeks apart. All but one respondent was female, 46 (77%) were married or in a defacto relationship, half (*n* = 33, 55%) were from two children households, 13 (22%) had a university degree, and two (3%) had Year 10 schooling or less. Forty-six (77%) were working full-or part-time, and 20 (33%) held a government health care card. It is not possible to calculate response rates for parents at both schools as questionnaires were posted to parents by the school and exact numbers posted are unknown.  Table 3 shows the outcomes for each questionnaire and the ICC (95% confidence interval) for each score. All ICCs were statistically significant and ranged from 0.37 to 0.92 with eight of the fourteen greater than 0.7. Cronbach's alpha varied from 0.13 to 0.78 for the scores from the parent questionnaire, with three of seven scores having alpha values greater than 0.5.

### 3.2. Teacher Questionnaire Test-Retest Reliability

Twenty-eight teachers (School 1: 24, School 2: 4) completed the teacher questionnaire twice. This is a response rate of 100% at School 1 and 50% at School 2. Most (*n* = 23) were female, and a majority taught across multiple year levels with half the respondents teaching in each of Reception to Year 5 and approximately one third teaching Year 6. Thirteen respondents had been at their current school for four years or longer, and for eight, it was their first year at the school.  Table 4 shows the outcomes for each completion of the questionnaire and the ICC (95% confidence interval) for each score. All ICCs were statistically significant and ranged from 0.42 to 0.86 with five of the twelve greater than 0.7. Cronbach's alpha varied from 0.11 to 0.91 for the scores from the teacher questionnaire, with four of six scores having alpha values greater than 0.5.

## 4. Discussion

The purpose of this study was to determine the reliability of the parent and teacher questionnaires developed to evaluate the *ewba* Community Programs, two questionnaires that assess the healthy eating and physical activity environments of children. The parent questionnaire assesses child dietary intakes, parent knowledge of health-related recommendations, and attitudes about healthy behaviours. The teacher questionnaire assesses the degree to which teachers incorporate healthy eating and activity facets in their daily teaching regime and their skills, attitudes, and knowledge around healthy eating and physical activity. 

Overall the surveys showed good test-retest reliability with only one outcome in each of the parent and teacher surveys having an ICC of less than 0.5 (parent,—money, and teacher, knowledge (fruit)). The test-retest reliability of knowledge questions in both the parent and teacher surveys was generally lower than the other items, with ICCs between 0.4 and 0.6. One possible reason for this is the difficulty determining which recommendations to use. The commonly promoted message in South Australia is “Go for 2&5” which are the adult recommendations for fruit and vegetable intake, respectively, and a message that is actively used in schools. However, the AGHE recommends a minimum of one and three serves of fruit and vegetables, respectively, for 8–11 year olds and two and four serves, respectively, for 12–18 year olds [20]. The AGHE includes additional options according to varying eating patterns with serves above these minimums. The response options aimed to cover these varying recommendations. These different messages are a potential source of confusion which may mean that respondents are “guessing” the correct option and this in turn would be a source of retest error.

The internal consistency of the scores in the parent and teacher questionnaires was poor to moderate. It is recommended that Cronbach's alpha is greater than 0.7 [23]. Only one score from the parent questionnaire (non-core food) and three from the teacher questionnaire were in line with this recommendation; however, the value is affected by the number of items in the scale, and it is common to find low Cronbach's alpha values with scales with less than ten items [24]. Four scores from the parent questionnaire had between five and ten items and three scores had less than five items. In the teacher questionnaire, four scores had between five and nine items and two scores had less than five items. Hence, the low number of items in the scores could be a reason for the poor to moderate internal consistency observed. Furthermore, the parent and teacher scores were created by summing items that measure the goals of the *ewba *program, specifically reducing intake of noncore foods and sweetened beverages, reducing screen time, and increasing fruit and vegetable intake, water intake, and physical activity. Unlike other scales in the literature, we would not necessarily expect all items to correlate. For example children with a high intake of crisps would not necessarily have a high intake of lollies (both single items in the noncore food score). This could explain the lower than ideal Cronbach's alpha values for some of the scores.

Four parent questionnaires were identified in the literature that had similar ranges (or slightly better) of internal consistency and test-retest reliability observed in this study. The Home Environment Survey [25] is completed by parents and measures how supportive the home environment is of healthy eating and physical activity. Cronbach's alpha for four subscales ranged from 0.66 to 0.84- and test-retest reliability was high with ICC more than 0.75 for all scales. The Children's Dietary Questionnaire, measuring parent report of child eating patterns, had four subscales with Cronbach's alpha for fruit and vegetable and noncore food subscales ranging from 0.62 to 0.76 with ICC for test-retest reliability ranging from 0.51 to 0.90 which was concluded to be satisfactory [26]. The “Meals in Our Household” questionnaire measured parent report of six domains, including family meal structure and mealtime behaviours. Test-retest reliability was assessed using the Spearman correlation for the six domains, and this ranged from 0.80 to 0.95, while Cronbach's alpha ranged from 0.39 to 0.93 across the six domains [27]. Similarly, a questionnaire measuring constructs believed to predict fruit and vegetable consumption (in children, completed by parents) had Pearson correlation ranging from 0.61 to 0.84 and Cronbach's alpha from 0.31 to 0.91 [28].

A strength of this study is the report on two questionnaires with multiple scores/indexes that simultaneously measure diet and physical activity environments of children. Many evaluation tools measure healthy eating or physical activity, while these tools measure the two simultaneously. Additionally, these tools are unique because they focus on behaviours, environments and attitudes, all of which have been demonstrated as factors contributing to the obesity epidemic [5]. Hence these two tools have the potential to be used in the evaluation of obesity prevention programs and consequently contribute to the evidence about obesity prevention. The indexes also cover a broad range of domains relevant to healthy eating and activity across the home and school environment and use scores as a way of interpreting information and gaining an overall picture. Tools which simultaneously evaluate environments that children are exposed to at home and school, as well as the attitudes of parents and teachers and diet and activity behaviours of children, are lacking in the literature. The use of an internal consistency analysis in addition to testing test-retest reliability is a strength, as is the participation of parents and teachers from both metropolitan and regional schools.

A notable limitation of the study is the sample size. As previously mentioned, the recommendation for a test-retest reliability study is a sample size of 100 [29]. The sample size for the teacher questionnaire fell well short of this (*n* = 28), and the sample size for the parent questionnaire was also below this (*n* = 60). The sample size of teachers at School 2 was low (*n* = 4) because only teachers of classes participating in the *ewba *evaluation were asked to participate (*n* = 8). The low sample size has implications for interpreting the results of the study, in particular those for the teacher questionnaire, because a larger sample size results in a smaller confidence interval which means we can be more certain that the true reliability coefficient is close to that which has been calculated [29]. Hence, the reliability of the teacher questionnaires in particular should be interpreted with caution. Despite the recommendation of 100 as the sample size for a test-retest reliability study [29], the sample size for similar studies varies considerably in the literature and the sample size for the parent questionnaire falls within this range. Forty-four parents were used to assess reliability of the “Meals in Our Household” questionnaire [27] and 38 childcare directors completed the “Nutrition and Physical Activity Self-Assessment for Child Care” in a test-retest reliability study [30]. Other studies had a larger sample size to test test-retest reliability, including Gattshall et al. [25] who tested reliability of the “Home Environment Survey” with 156 parents and Randall-Simpson et al. [31] who had 140 parents participate to assess reliability of the “Nutrition Screening Tool for Every Preschooler.” In addition, while creation of scores can provide more meaningful and reliable results, important information may be missed or results misinterpreted without some secondary investigation of reliability of individual items such as in the parent attitudes to healthy eating and physical activity scores. A possibility for future research is to test the internal validity of the parent and teacher questionnaires and to retest the reliability of the teacher questionnaire with a larger sample size. 

## 5. Conclusions

The Parent and Teacher questionnaires for the *ewba *Community Programs are a moderately reliable method for assessing child intakes, environments, attitudes, and knowledge associated with healthy eating and physical activity. Similar tools are lacking in the literature. These questionnaires assess relevant information and the scores present this information in a meaningful manner, suggesting that they may be useful in similar settings to evaluate similar obesity prevention programs.

## Figures and Tables

**Table 1:** The 14 parent outcomes grouped into six categories, the individual items contributing to each score and the possible responses for each item.

Category score	Items in each score	No. of items	Response
Food availability			
Sweet beverages	Purchasing frequency of diet soft drinks*; regular soft drinks*; fruit juice/drink*; cordial*.	4	Never, rarely, monthly, fortnightly, weekly, or more (assigned 1 to 5, resp.)
Noncore foods	Purchasing frequency of non-core foods*: crisps or similar snacks*; cakes, muffins, and donuts*; savoury biscuits*; sweet biscuits*; muesli bars or fruit bars*; lollies*; chocolates*.	7	Never, rarely, monthly, fortnightly, weekly, or more (assigned 1 to 5, resp.)
Fruit and vegetables	Purchasing frequency of vegetables, fresh fruit; dried fruit.	3	Never, rarely, monthly, fortnightly, weekly, or more (assigned 1 to 5, resp.)

Healthy eating scores			
Healthy eating attitudes	Fresh fruit and vegetables are too expensive; I find it difficult to get my child to eat fruit every day; I find it easy to get my child to eat vegetables every day*; my child eating fruit and vegetables every day is a high priority for me*; there are no stores selling fresh fruit and vegetables within 10 minutes walking distance of our home; there is enough information about healthy eating for children in our neighbourhood or school*.	6	Strongly agree; agree; not sure; disagree; strongly disagree
Healthy eating rules	Did the child(ren) watch TV at meal times?* Did the child(ren) have a set meal routine? Were the child(ren) encouraged to eat fruit? Were the child(ren) encouraged to eat vegetables? Were the child(ren) offered dessert as a reward for eating less favourite foods?* Were limits set on the types of foods they can snack on regularly? Were limits set on the types of drinks the children can drink regularly? Were the children reminded to drink water? Did an adult sit down with the children when they ate meals? Did you make something else if the children did not like what was being served?*	10	Never, rarely, sometimes, often, always

Healthy eating child behaviour			
Fruit	Serves of fruit consumed each day by child.	1	1,2, 3,4, 5, or more serves per day
Vegetables	Serves of vegetables consumed each day by child.	1	1,2, 3,4, 5, or more serves per day
Water	Drink child selects when thirsty.	1	Cordial, fruit juice, fruit drink, soft drink, milk, and water

Knowledge of healthy eating			
Fruit	Recommended serve fruit.	1	Less than 1, 1-2, 3–5 and more than 5 per day
Vegetables	Recommended serve vegetables.	1	Less than 1, 1-2, 3–5 and more than 5 per day

Activity scores			
Activity attitude	It is not safe for primary school-aged children to walk or cycle alone in our neighbourhood during the day; my child being physically active on most days of the week is *not* a high priority for me; our closest park/playground from home is safe for primary school-aged children to play in*; there are enough recreation/sports facilities (e.g., tennis courts, skateboard ramp) in our neighbourhood to encourage primary school-aged children to be physically active*; there are playgrounds, parks, and/or ovals within easy walking distance of our home*; there is enough information about physical activity opportunities for children in our neighbourhood or school*; there is enough variety of equipments at home for children to use to be physically active (e.g.; balls, skipping ropes)*.	7	Strongly agree; agree; not sure; disagree; strongly disagree
Activity rules	Were the children encouraged to play outdoors? Were limits set on the amount of time the children can watch TV? Did adult family members walk or cycle to get to or from places (e.g., work, shops)?	3	Never, rarely, sometimes, often, always

Ungrouped			
Money	Have run out of food in the last 12 months and did not have the money to buy more.	1	Strongly agree; agree; not sure; disagree; strongly disagree
Breastfeeding	Difficult for women in our neighbourhood to breastfeed in public places.	1	Strongly agree; agree; not sure; disagree; strongly disagree

*Items reverse scored.

**Table 2:** The 12 teacher survey outcomes grouped into three categories, assessing child exposure, teacher skills and attitudes, and teacher knowledge, the individual items contributing to each score and the response for each item.

Category score	Items in each score	No. of items	Response
Child exposure			
Healthy eating (HE)	Classroom lessons about HE; homework about HE; HE embedded into curriculum in 2005 or 2009; HE is a priority in this school.	4	Not used, less than once a term, once a term; weekly; daily None, little, some, a lot Strongly agree; agree; not sure; disagree; Strongly disagree
Fruit and vegetables	Fruit and vegetable cooking sessions; fruit and vegetable tastings; growing fruit/vegetable; visits to fruit/vegetable growers; fruit/vegetable breaks.	5	Not used, less than once a term, once a term; weekly; daily
Physical activity (PA)	Teaching fundamental movement skills; PA during class; homework based on PA; PA used in curriculum in 2005 or 2009; PA used to teach other areas of curriculum in 2005 or 2009; PA is a priority in this school; enough facilities to encourage kids to be active at break times; school offers wide variety of physical activities.	8	Not used, less than once a term, once a term; weekly; daily None, little, some, a lot Strongly agree; agree; not sure; disagree; strongly disagree
Water	Encouraging water bottles.	1	Not used, less than once a term, once a term; weekly; daily

Teacher skills/attitude			
Healthy eating	Have HE training or expertise; (Received HE professional development in 2004-2005)^#^; HE training 2006 and 2007; HE training 2008 and 2009; HE programs or resources used in 2005 or 2009.	4^#^	None, little, some, a lot Yes, no
Fruit and vegetables	Motivated to teach students the importance of eating fruit and vegetable every day; unsure of my ability to teach students the importance of eating fruit and vegetable;* encouraging kids to eat fruit and vegetables is the responsibility of the family;* little support from external organisations to promote fruit and vegetable in 2005/2009;* kids who eat more fruit and vegetable behave better; important for teachers to role model eating fruit and vegetable.	6	Strongly agree; agree; not sure; disagree; strongly disagree
Physical activity	PA training or expertise; (PA professional development in 2004-2005)^#^: PA training 2006 and 2007; PA training 2008 and 2009; used PA programs or resources in 2005/2009; active lunch time play is important; not important for teachers to role model being physically active*; it is better for students to walk or cycle to school; little support from external professionals to promote PA in 2005/2009'*; confident in ability to include PA opportunities for kids in the classroom.	9^#^	None, little, some, a lot Yes, no Strongly agree; agree; not sure; disagree; strongly disagree
School environment	School encourages its PA facilities to be used by the wider community.	1	Strongly agree; agree; not sure; disagree; strongly disagree

Teacher knowledge			
Fruit	Serves of fruit a primary school-aged child needs daily.	1	Select from <1, 1-2, 3–5, >5, unsure
Vegetables	Serves of vegetables a primary school-aged child needs daily.	1	Select from <1, 1-2, 3–5, >5, unsure
Physical Activity	Minutes of PA needed daily by primary school-aged child.	1	None, 30 minutes, 60 minutes, 90 minutes, unsure
Television	Maximum daily screen time recommended for children.	1	1 hour, 2 hours, 3 hours, 4 hours, unsure

^#^“HE PD/PA training in 2004-2005” item only collected at baseline, replaced by two items covering 2006/7 and 2008/9 in reliability study, thus one less item in each score at baseline.

*Items reverse scored.

HE: healthy eating.

PA: physical activity.

**Table 3:** Reliability (*n* = 60^†^) demonstrated by intraclass correlation coefficient (ICC) and 95% confidence intervals (CI) for the parent outcomes and internal consistency for the parent scores.

Score	Reliability data			Median (interquartile range)		Target score/response
	ICC	95% CI	Cronbach's alpha	Time 1		
Food availability						
Sweet beverages	0.86**	0.76–0.91	0.60	13 (10–15)	12.5 (10–15)	≥12 (less than weekly each item)
Non-core foods	0.81**	0.7–0.89	0.78	20 (17–23)	20 (17–23)	≥21 (less than weekly each item)
Fruit and vegetables	0.61**	0.42–0.75	0.13	13 (12–14)	13 (12–14)	≥12 (weekly or more often each item)

Healthy eating						
Healthy attitudes	0.55**	0.34–0.70	0.35	22 (19–24)	22 (20–24)	≥24 (agree/strongly agree each item)
Healthy rules	0.77**	0.63–0.86	0.55	42 (39–45)	42 (38–45)	≥40 (often/always each item)

Healthy eating behaviour						
Serves of fruit	0.80**	0.69–0.88	N/A	2 (1-2)	2 (1–3)	2 or more^§^
Serves of vegetables	0.86**	0.77–0.91	N/A	3 (1–4)	3 (2–4)	3 or more^§^
Drink when thirsty	0.92**	0.88–0.95	N/A	6 (6-6)	6 (5-6)	Water^§^

Knowledge of healthy eating						
Fruit	0.61**	0.41–0.75	N/A	2 (2-2)	2 (2-2)	1-2/day^§^
Vegetable	0.66**	0.49–0.78	N/A	3 (2-3)	3 (2-3)	3–5 serves per day^§^

Activity						
Attitude activity	0.74**	0.59–0.83	0.46	24 (22–28)	26 (22–28)	≥28 (agree/strongly agree each item)
Rules activity	0.67**	0.50–0.79	0.21	11 (9–12)	11 (9–12)	≥12 (often/always each item)

Other						
Money	0.37*	0.13–0.54	N/A	2 (2-2)	2 (2-2)	≥3 (disagree/strongly disagree)
Breastfeeding	0.71**	0.56–0.82	N/A	2 (2-2)	2 (2-2)	≥3 (disagree/strongly disagree)

*P < 0.05.

**P < 0.001.

^†^ *n* varied from 57 to 60 with *n* for sweetened beverage score 53.

^§^As per Australian Guide to Healthy Eating [15].

N/A: not applicable.

**Table 4:** Reliability demonstrated by intraclass correlation coefficient (ICC) and 95% confidence intervals (CI) for the teacher outcomes and internal consistency for the teacher scores.

Score	Reliability data			Median (interquartile range)		*N*	Target score/response
	ICC	95% CI	Cronbach's alpha	Time 1	Time 2		
Child exposure							
Healthy eating	0.86**	0.67–0.94	0.61	9 (6–10)	9 (7–12)	20	≥15 (weekly, some, agree as relevant)
Fruit and vegetables	0.77**	0.52–0.90	0.91	7.5 (5–11)	7 (5–13)	22	≥16 (weekly each item)
Physical activity	0.67**	0.38–0.84	0.58	21.5 (19–26)	22 (19–25)	23	≥30 (weekly, some, agree as relevant)
Water	0.82**	0.63–0.91	N/A	5 (4-5)	5 (5-5)	26	Used daily

Teacher skills/attitudes							
Healthy eating	0.71**	0.40–0.88	0.60 (*n* = 24)	4 (4–6)	5 (5-5)	24	≥7 (some, yes as relevant)
Fruit and vegetable	0.61**	0.30–0.80	0.11	23 (21–24)	22 (22-23)	26	≥24 (agree each item)
Physical activity	0.56*	0.11–0.82	0.22 (*n* = 23)	25 (24–27)	24 (23–26)	16	≥27 (some, yes, agree as relevant)

School environment							
	0.67**	0.39–0.84	N/A	3 (2-3)	3 (2-3)	26	Agree

Teacher knowledge							
Fruit	0.42*	0.05–0.67	N/A	2 (2-3)	2 (2-3)	27	1-2 serves
Vegetable	0.63**	0.33–0.81	N/A	3 (2-3)	3 (3-3)	27	3–5 serves
Screen time	0.53*	0.20–0.76	N/A	2 (1-2)	2 (2-2)	27	2 hours
Physical activity	0.81**	0.63–0.91	N/A	3 (2-3)	2 (2-2)	27	60 minutes

*P < 0.05.

**P < 0.001.

N/A: not applicable.
